# The Impact of Estimated Energy and Protein Balances on Extrauterine Growth in Preterm Infants

**DOI:** 10.3390/nu15163556

**Published:** 2023-08-11

**Authors:** Ioanna Lygerou, Stavroula Ilia, Panagiotis Briassoulis, Anna Manousaki, Marina Koropouli, Eleftheria Hatzidaki, George Briassoulis

**Affiliations:** 1 Postgraduate Program “Emergency and Intensive Care in Children Adolescents and Young Adults”, School of Medicine, University of Crete, 71003 Heraklion, Greece; manousaki2008@gmail.com (A.M.); briasoug@uoc.gr (G.B.); 2 Department of Neonatology/Neonatal Intensive Care Unit, University Hospital of Heraklion, School of Medicine, University of Crete, 71500 Heraklion, Greece; el.hatzidaki@uoc.gr; 3 Pediatric Intensive Care Unit, University Hospital, School of Medicine, University of Crete, 71110 Heraklion, Greece; briaspan@med.uoa.gr; 4 Attikon University Hospital, School of Medicine, National and Kapodistrian University of Athens, 12462 Athens, Greece; 5 Neonatal Intensive Care Unit, Venizelio General Hospital, 71409 Heraklion, Greece; koropouli.mar@gmail.com

**Keywords:** preterm, nutrition, growth, neonatal, energy, protein, weight z-scores, birthweight, enteral, parenteral

## Abstract

Background: Nutritional support of preterm infants remains a field of debate in the literature and clinical practice varies significantly. Adequate nutrition should promote growth and aim for optimal later neurodevelopment. However, it is often impaired by prematurity-associated morbidity and the physiologic immaturity of preterm infants. This study assessed the impact of energy and macronutrient provision on growth velocity and outcome and explored differences attributed to the heterogeneity of the preterm population. Methods: We retrospectively collected clinical and nutritional data from neonates hospitalized in two separate Neonatal Intensive Care Units (NICUs). Estimated energy and protein balance were calculated based on the ESPGHAN guidelines and their association with the growth outcome was explored. Growth assessment was based on somatometry Delta (Δ) z-scores at discharge. Results: In total, 174 neonates were included in the study. By day 14, most preterm infants were exclusively enterally fed, whereas there were infants in the <28 and 28–31^+6^ subgroups fed exclusively parenterally. Energy balance was positive for all gestational age (GA) subgroups except for those born <28 weeks. Protein balance was consistently positive for extremely premature but negative for late preterms. Cumulative substrates provisions were strong predictors of a positive energy or protein balance in the <34 weeks GA preterms on days 14 (ROC analyses, *p* < 0.001) and 7 (*p* < 0.05). A higher GA (*p* = 0.013) and enteral nutrition (*p* = 0.005) were additional predictors of a positive energy balance. All GA subgroups had a negative Δ z-score of weight at discharge. In the <34 GA subcohorts, a positive protein balance on day 14 (*p* = 0.009) and a short time to regain birth weight (exp(B) 3.1 (*p* = 0.004)) were independently associated with a positive Δ z-score of weight at discharge. Conclusions: Early achievement of a positive energy and protein balance, based on the ESPGHAN guidelines, is crucial to ensure optimal postnatal growth and prevent extrauterine growth restriction, a relatively common occurrence in preterm infants.

## 1. Introduction

Preterm birth is a nutritional emergency. During the third trimester of pregnancy, the foetus undergoes a period of rapid growth and brain development. Abrupt interruption of the placental supply of micro- and macronutrients exposes the premature infant to the risk of malnutrition [1]. These infants are born with low stores of key-nutrients, have poor thermoregulation and frequently experience prematurity-associated morbidity such as respiratory distress syndrome and sepsis, all contributing to their high energy requirements. However, despite their structurally and functionally immature gut, which might slow feeding advancement and delay the time it takes to reach full feeds [2,3], many critically ill infants may receive enteral nutrition far before term-equivalent age [4].

Despite significant advances in perinatal care in recent years, growth faltering and extrauterine growth restriction remain an issue of significant importance, especially for those born extremely premature with very low birthweight (VLBW) [5]. Traditionally, postnatal growth rates analogous to intrauterine rates have been considered the optimal goal proposed by the American Academy of Paediatrics in 1977 [6]. In that spirit, widely used tools for monitoring postnatal growth, such as the Fenton growth curves, were constructed. These curves are based on size at birth measurements and allow the longitudinal plotting of postnatal growth of preterm infants and the comparison with their in utero counterparts. Recently published, the INTERGROWTH-21st standard curves are based on the concept that postnatal growth should follow the growth of the “healthy” preterm of the same age rather than that of the foetus. These curves are prescriptive tools following the WHO’s child growth standards and reflect how a preterm infant should grow given the optimal conditions [7,8]. Since their publication, a number of studies have shown reduced incidence of extrauterine growth restriction (EUGR) when their use is implemented [9,10].

A few studies have tried exploring the impact of macronutrient provision during the first days of life on the extrauterine growth outcome. The early phase of parenteral nutrition in very preterm infants is followed by a transition phase characterized by de-escalation of parenteral nutrition and escalation of enteral feeds, gradually proceeding to full enteral nutrition. Ideally, the mother’s own milk, or donor human milk, is recommended as the first choice for feeding preterm infants because it contains biological components that compensate for organ systems immaturity [11]. However, human milk (HM) of mothers who give birth to very preterm infants is protein-deficient [12], resulting in accumulated nutrient deficits, increasing the possibility of growth restriction and poor neurodevelopment [13]. Even short periods of undernutrition can adversely impact brain growth [14]. During the transition phase, inadequate cumulative energy, protein, fat, and carbohydrate intakes were associated with weight and head circumference growth restriction [15]. Because usual volumes of human milk do not provide the recommended macro- and micronutrients to meet the specific needs of VLBW infants, it is recommended that HM fortifiers are added to enrich their nutrient content and better promote growth. Ideally, human milk analyzers can be used for an individual fortification approach [16,17].

Past studies have reported the association of adequate energy and protein provision with positive neurocognitive outcome [18,19]. Quantifying the association between growth and nutrition intake, it was shown that each 10 kcal/kg/d and 1 g/kg/d macronutrient increase were independently associated with increased weight, length, and head circumference over 7 days [20]. To prevent nutritional deficits in human milk-fed preterm infants, individualized fortification of human milk based on measuring its macronutrient content has also been suggested for preterms born in less than 33 weeks [21]. On the other hand, higher nutritional substrates during the first weeks of life of VLBW infants were shown to predict energy balance after 20 years [22]. Rapid weight gain and catch-up growth in the early postnatal period has been implicated in later cardiometabolic risk [23]. However, not only nutritional factors, but also anthropometric, demographic, clinical characteristics, and morbidity were independently associated with VLBW infant growth [24].

Given the diversity of the preterm population and the varied nutritional protocols among Neonatal Intensive Care Units (NICUs), results on the growth outcome in various gestational age (GA) groups of preterm infants have been inconclusive. Publication of nutritional guidelines has aimed to bridge this gap, but clinical practice remains largely empirical. The updated European Society for Paediatric Gastroenterology Hepatology and Nutrition Committee on Nutrition (ESPGHAN CoN) consensus-based recommendations on nutritional management of preterm infants accepts that there is a lack of strong evidence for most nutrients and topics [25]. In our study, we gathered clinical and nutritional data for a cohort of infants of all gestational ages born and hospitalized in two separate NICUs. Our aim was to record the applied nutritional practices and correlate pertinent indices, such as the longitudinal energy and protein balance, based on ESPGHAN’s latest guidelines, with growth outcome in all GA groups; and secondly, to analyse the correlation of multiple enteral and parenteral nutritional characteristics in separate GA subgroups with demographic, anthropometric, and clinical data. 

## 2. Materials and Methods

### 2.1. Study Design and Population

This is a retrospective, two-centre study conducted in the Neonatal ICUs at the Heraklion University Hospital (HUH) and its twin NICU in the Heraklion City, Venizeleio General Hospital (VGH), Heraklion, Crete. Our study population consisted of neonates admitted in the NICU from July 2019 to January 2020 for VGH and from December 2020 to May 2021 for HUH, two consecutive periods following the same feeding protocol in the two units. Accordingly, the data were pooled (identical populations, identical patient data collected, unique protocols used). The research protocol received approval from both hospitals’ scientific and Ethics committee (approval IDs: 14408/3-8-2020 and 8199/17-5-2021). Due to the retrospective nature of the study, consent was waived. We included neonates of all gestational ages admitted on the day of birth and hospitalized for 3 or more consecutive days. We excluded neonates with congenital birth defects or syndromes impairing enteral feeding, and those with severe perinatal asphyxia. All data were collected from patients’ medical records using a predefined form that included demographic, clinical, anthropometric, and nutritional information.

### 2.2. Clinical Data

Our population was divided according to gestational age in the following subgroups: extremely preterm (≤28 weeks), very preterm (28–31^+6^ weeks), moderately preterm (32–33^+6^ weeks), late preterm (34–36^+6^ weeks), and term infants (≥37 weeks). Infants whose birthweight was on or below the 10th percentile for GA were further classified as small for gestational age (SGA). This is a statistical definition that reflects a deviation from the average foetal growth and is commonly used in clinical practice and in the literature to group and study these patients [26]. We used INTERGROWTH-21st set of standard curves for newborn size at birth [8]. Intrauterine growth restriction (IUGR), or fetal growth restriction (FGR), is defined as a deceleration of the anticipated foetal growth rhythm due to either foetal, maternal, or placental aetiology. IUGR is therefore diagnosed by ultrasound antenatally, and these neonates are not necessarily SGA [9,26]. In our population, we were based on the antenatal records of the neonates to categorize them as IUGR.

The type and duration of respiratory support of neonates with Respiratory Distress Syndrome (RDS) were recorded. Infants initially intubated and invasively mechanically ventilated (IMV) and then weaned on non-invasive mechanical ventilation (NIMV) were counted in the IMV category. Respiratory support in the form of nasal continuous positive airway ventilation (nCPAP) was also used in some cases of transient tachypnoea of the newborn, a condition clinically and radiologically different from RDS.

Other major clinical incidents we recorded were bronchopulmonary dysplasia, necrotizing enterocolitis, sepsis (early and late onset), and surgery (congenital malformations or acute events). These events are described as discharge diagnoses, and we verified them per the patients’ medical records.

We also included a risk assessment score, the Clinical Risk Index for Babies (CRIB) II severity score. This is a 5-item tool that predicts mortality and risk for death in infants born less the 32 weeks gestational age [27].

### 2.3. Anthropometry

Weight, length, and head circumference were measured at birth and discharge. Weight was measured daily using digital scales (SCALA model 727, Hamburg, Germany). Neonates in incubators were weighed daily in their incubator scales. Length and head circumference were measured weekly using non-stretchable tape (SECA, Hamburg, Germany). Percentiles and z-scores were extracted using INTER-GROWTH-21st set of standard curves available at their website [28,29]. Postnatal weight gain velocity can be demonstrated using either numerical calculations (g/kg/d, g/d) [30] or difference in z-score between two timepoints. There is no uniform method used, such that the comparison between similar studies is difficult. The most frequent method in recent literature is the z-score change over time, based on intrauterine growth charts, such as the Fenton curves [31]. In our study, we used the change in z-score between admission (birth) and discharge for all anthropometry parameters. Instead of intrauterine growth curves, we used the INTERGROWTH-21st set of standard curves for postnatal growth of preterm infants. This shift in conventional methodology has been recently proposed based on the notion that preterm infants should grow according to the “healthy preterm” and not the healthy fetus counterpart [7].

### 2.4. Nutritional Data

Nutritional data were collected on the 3rd, 7th, and 14th day for all GA subgroups. We recorded the type of feeding (enteral, parenteral, or mixed), the timing (day of initiation) and duration of parenteral feeding. For parenterally fed infants, the type of intravenous access (central or peripheral catheter) as well as potential complications, mechanical or biochemical, were recorded. For enterally fed infants, we collected information regarding the method of feeding (nasogastric tube, bottle, or breastfeeding) and reported complications of enteral feeds, including necrotising enterocolitis (NEC).

The two NICUs provided the similar formulas (term and preterm) for enterally fed infants (Supplemental Table S1), as well as human milk fortifiers in powder form (Supplemental Table S2). Parenteral solutions in the two NICUs do not come in pre-made bags; they are made on a daily basis based on the physicians’ prescription. The composition of amino acid solutions and lipid emulsions used can be found in the Supplemental Materials (Supplemental Table S3).

Enteral nutrition protocol in the two NICUs follow ESPGHAN’s general principles outlined in the recent position paper [25]. In hemodynamically stable preterm infants, trophic feeding (volumes < 24 mL/kg/d) starts preferentially in the first 24 (48 if not possible) hrs of life. Advancement of enteral feeds follow increments of 18–30 mL/kg/d if the infant remains stable and tolerates it. Feeds are given as boluses via nasogastric tube, or by mouth in older infants. The ultimate fluid volume target in exclusively enterally fed infants is 150–180 mL/kg (Supplemental Figure S3).

We calculated the intakes of energy (in kcal/kg body weight) and carbohydrates, fat, and protein (in g/kg body weight) on the 3rd, 7th, and 14th day. Total daily nutrient intakes included parenteral and enteral intakes. For parenteral nutrition, these calculations were based on the prescribed quantity and composition of fluids on the same day. Due to the lack of milk analysers, the exact composition of nutrients in human milk could not be accurately determined. To mitigate this for enterally fed infants, our calculation was an approach based on published concentrations of macronutrients in human milk and infant formula [32]. We distinguished two categories, term and preterm milk (or formula), given the fact that preterm formula and human milk from mothers who deliver prematurely is higher in all macronutrients, especially protein [33]. An average of macronutrient concentrations of term human milk and term formula was calculated (respectively, for preterm milk and formula, as shown in Table 1). Infants were assigned to each category depending on the prescribed formula (preterm/term).

To calculate the energy and protein balances (estimated), we based the calculations on two recent position papers by ESPGHAN published in 2021 and 2023, regarding the nutritional management of the critically ill neonate [34] and preterm infants < 1800 g [25]. In the work by Moltu et al., it is proposed that nutritional support should be individualized depending on the phase of critical illness. We adopted this approach by setting the minimum requirements of energy and protein provision for the “recovery” phase, which theoretically begins from day 7 and onwards (Table 2). Balances were calculated by subtracting the intake from the minimum required amount of either protein or energy. For infants who were receiving parenteral and trophic enteral feeds, we set the minimum requirement of parenteral feeds which is lower. In the case of simultaneous parenteral and enteral nutrition, the higher enteral energy needs were used for calculations.

### 2.5. Statistical Analysis

The Shapiro–Wilk test was used to assess the normality of the distribution. The descriptive data are reported as means and standard deviation (SD) or median and interquartile range (IQR) in case of skewed distributions or as frequencies and percentages when appropriate. Comparisons among groups for quantitative variables were carried out by one-way ANOVA with Bonferroni’s honestly significant differences (HSD) as a post hoc test for parametric data. The independent-samples Kruskal–Wallis one-way analysis of variance by ranks across groups was used for nonparametric data, adjusted by the Bonferroni correction for multiple tests. Comparisons between categorical variables were conducted using the chi-square (*χ*^2^) test. Spearman’s correlation coefficient was used for correlation between two continuous variables. Paired differences for continuous variables in the same subjects were analysed using the related samples of Friedman’s two-way analysis of variance by ranks. Pairwise comparisons between pairs of anthropometric z-scores upon admission and at discharge were analysed using the Wilcoxon signed-ranks test, adjusted by the Bonferroni correction for multiple tests. A linear regression model (backward method) was adopted to examine whether any of the recorded anthropometric, clinical, and metabolic variables are independently associated with the days on invasive mechanical ventilation or the mean length of stay (LOS). To evaluate factors affecting energy and protein balance, the areas under the receiver operating characteristic curves (AUROCs) for nutritional variables (type of feeding, substrates) and anthropometric and clinical variables significantly predicting a positive energy or protein administered—recommended energy and protein difference at day 14 were calculated in the whole group and in the small preterm subcohorts. To evaluate factors affecting growth, the AUROCs for variables significantly predicting a positive Δ z-score of weight at discharge were calculated in the whole group and in the small preterm subcohorts. A binary logistic regression analysis model (backward LR) was adopted to examine independent associations of nutritional and non-nutritional variables with a positive Δ z-score of weight at discharge in the whole group and in the small preterm subcohorts. All tests were two-tailed, and the threshold for statistical significance was set to *p* < 0.05. Statistical analyses were performed using SPSS Version 29 (IBM SPSS Statistics, Chicago, IL, USA) and GraphPad Prism Version 9.0 (GraphPad Software, Inc., San Diego, CA, USA).

## 3. Results

### 3.1. Demographic, Anthropometry, and Clinical Data

In total, 174 neonates were included in the study, most being boys (61.5%, *n* = 107). Ethnicity was almost exclusively Greek (97%). Multiple births (twin or triplet) accounted for 33.9% (*n* = 59) of the studied neonates, while a history of assisted reproduction was positive in 51 cases (29.3%). Caesarean section was the prevalent delivery method (*n* = 147, 84.5%). The mean mother’s age was 32.8 years.

Neonates of all gestational ages were included, with the median GA being 34 weeks. The largest subgroup was that of late preterm infants born between 34–36^+6^ weeks, comprising 44.3% (*n* = 77) of the total population (Supplemental Figure S1A). The mean weight on admission was 2080 ± 651 gr. Preterm infants < 1500 gr comprised 14.9% of the population (Supplemental Figure S1B). Small for gestational age infants, i.e., infants whose birthweight was below the 10th percentile for age, accounted for 18.4% of the population. All but one neonate of the study survived, and the CRIB II severity score was significantly higher in smaller GA (*p* < 0.001) (Table 3).

Aside from prematurity, respiratory distress syndrome was the predominant cause of admission. Nasal CPAP was the mainstay of respiratory support. The smallest preterm neonates were more often supported by invasive mechanical ventilation (IMV).

Caesarean sections (85%) were increased among cases of multiple gestation (100%) and assisted reproduction (98%), and in the late preterms, the 28–31^+6^ GA, the SGA, BPD, and sepsis subcohorts, approaching a prevalence of 90%, compared to 80% of all other groups (*p* < 0.001).

In a linear regression model (backward method), days on invasive MV were inversely independently associated with gestational age (beta −0.66, *p* < 0.001). The mean length of stay was 22.78 ± 20.6 days and was inversely associated with the gestational age (beta −0.83, *p* < 0.001) and birthweight (beta −0.71, *p* < 0.001) (Supplemental Figure S2). Nutritional markers were not independently associated with MV duration or LOS.

### 3.2. Nutritional Data

**Figure 1:**
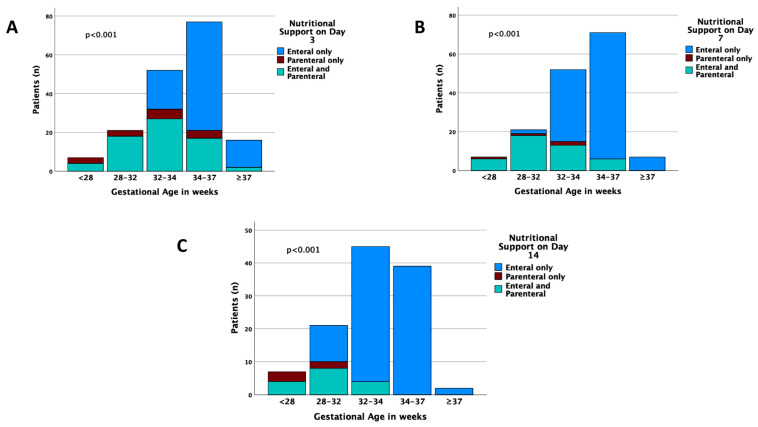
Type of nutritional support across gestational ages on Days 3 (**A**), 7 (**B**), and 14 (**C**). The *p*-values express differences among all age groups.

Type of nutritional support between GA subgroups on days 3, 7, and 14 are shown in Figure 1.

By day 14, most preterm infants were exclusively enterally fed, whereas there were infants in the <28 and 28–31^+6^ subgroups fed exclusively parenterally. Time to initiate enteral nutrition was longer in smaller GA (*p* < 0.001) (Table 4). Usual barriers to initiating and advancing enteral feeding in our extremely or very small preterms were hemodynamic instability, emesis or surgical problems, and the clinician’s reluctance because of suspected NEC.

**Figure 2:**
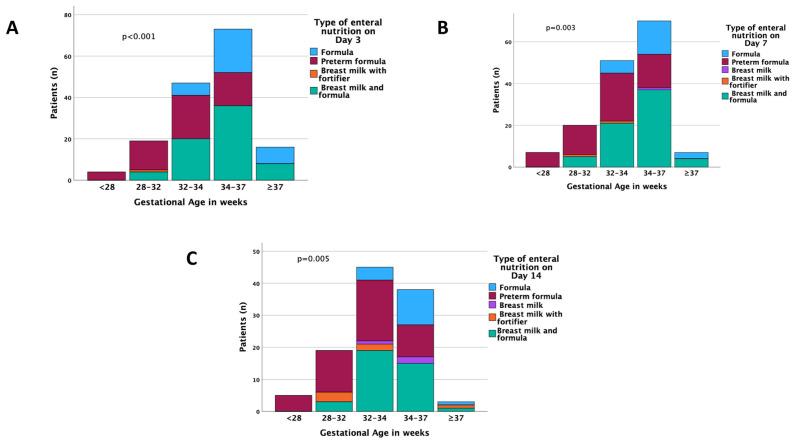
Longitudinal distribution of various types of enteral feeding across gestational ages on Days 3 (**A**), 7 (**B**), and 14 (**C**). The *p*-values express differences among all age groups.

Duration of parenteral feeds differed significantly between different gestational ages (*p* < 0.001). For the total population, parenteral feeding started within 1.32 ± 2.6 days. Most enterally fed infants were fed preterm formula, with or without breast milk (Figure 2).

Only a minority of preterms were fed exclusively by breast milk, with or without fortifier. Complications from enteral feeding were recorded in 57 (32.8%) patients, the most frequent being vomiting (*n* = 28, 49.1%) and gastric residue (*n* = 14, 24.6%). One patient in the extreme premature subgroup developed NEC. Biochemical complications during parenteral nutrition were recorded in 26 (23.2%) cases, the most frequent being hypocalcaemia (69.2%) and hypokalaemia (15.4%).

### 3.3. Energy and Macronutrients

Cumulative energy (kcal/kg) from enteral and parenteral feeding for the sum of neonates increased significantly from 79.2 ± 19.9 kcal/kg on Day 3 to 111.9 ± 31.4 kcal/kg on Day 7 and to 120.2 ± 31.8 kcal/kg on Day 14 *(p* < 0.001). The cumulative energy differed among age groups during the study period and between days in all preterm groups (Table 5). Each day cumulative energy data, collected on a subset of infants, did not produce any significant results compared with the three different day comparisons (Supplemental Table S4).

Cumulative protein (g/kg) from enteral and parenteral feeding for the sum of neonates increased significantly from 2.4 ± 0.9 g/kg on Day 3 to 3.1 ± 1 g/kg on Day 7 and to 3.3 ± 0.8 g/kg on Day 14 *(p* < 0.001). The cumulative protein differed among age groups during the study period and between days in the 32–34 and 34–37 GA groups (Table 5). Each day, cumulative protein data, collected on a subset of infants, did not produce any significant results compared with the three different day comparisons (Supplemental Table S4).

**Figure 3:**
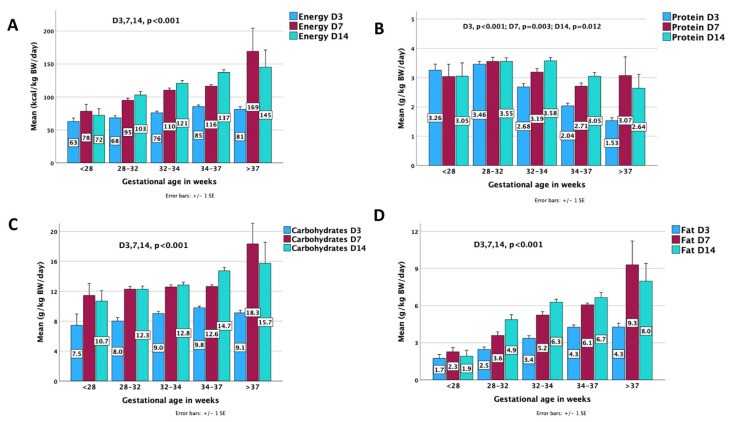
Cumulative distribution of provided (**A**) energy, (**B**) protein, (**C**) carbohydrates, and (**D**) fat through enteral and parenteral routes across gestational ages on days 3, 7, and 14. Substrates are expressed in g/kg/day and energy in kcal/kg/day. The *p*-values express differences among all age groups at different days (D3, D7, D14); BW = Body Weight; D = Day.

Macronutrients provided through the enteral and parenteral route were escalated by GA, except protein, which was higher in the 28–32 GA group (*p* < 0.001). Comparative substrate and energy provided on days 3, 7, and 14 in the different GA groups are presented in Figure 3.

### 3.4. Estimated Energy and Protein Balance

Estimated energy balance, expressed as the difference of administered total energy minus the recommended by ESPGHAN guidelines energy for enterally and parenterally fed infants, was positive for all preterm subgroups on Days 7 and 14 except for the extremely premature, who maintained a negative energy balance based on the minimum energy requirements of ESPGHAN guidelines (Table 6).

Protein balance, expressed as the difference of administered total protein minus the recommended by ESPGHAN guidelines protein requirements for enterally and parenterally fed infants, was positive on Day 7 for infants < 32 weeks GA and full-term infants. In contrast, moderate and late preterm infants demonstrated a negative protein balance on Day 7, which was reversed in moderate preterms on Day 14 but remained negative for late preterms (Table 6). Furthermore, the 28–31^+6^ subgroup, despite the initially positive protein balance, had a negative balance on Day 14.

### 3.5. Predictors of Estimated Energy or Protein Balance

#### 3.5.1. Predictors of a Positive Estimated Energy Balance

**Figure 4:**
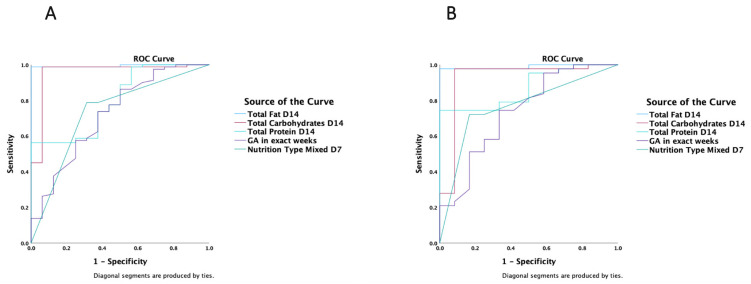
ROC analysis of a positive estimated energy balance on day 14 including (**A**) all gestational age (GA) subcohorts; (**B**) the smallest preterm infants with GA < 34 weeks; nutrition type indicates a lower to higher scale: enteral vs. parenteral vs. enteral and supplemental parenteral.

In a ROC analysis, total (enteral and parenteral) fat (AUC 0.99 (95%CI 0.98–1.0), *p* < 0.001), carbohydrate (AUC 0.96 (95%CI 0.89–1.0), *p* < 0.001), and protein (AUC 0.80 (95%CI 0.69–0.91, *p* < 0.001) intakes on day 14, a higher GA (AUC 0.72 (95%CI 0.58–0.87), *p* = 0.005) and mixed rather than enteral nutrition (AUC 0.73 (95%CI 0.59–0.87), *p* = 0.004), were all strong predictors of a positive energy balance on day 14 (Figure 4A). The type of delivery, the presence of assisted reproduction, SGA, IUGR, and weight on admission did not influence the energy balance on day 14 (Supplemental Table S5). Similar results for macronutrients (*p* < 0.001) and enteral nutrition (*p* = 0.02) predictive ability were obtained when the analysis was run on day 7.

When the ROC analysis was restricted to subcohorts with GA < 34 weeks, total (enteral and parenteral) fat (AUC 0.99 (95% CI 0.96–1.0), *p* < 0.001), carbohydrate (AUC 0.92 (95% CI 0.81–1.0), *p* < 0.001), and protein (AUC 0.87 (95% CI 0.78–0.97, *p* < 0.001) intakes on day 14, and a higher GA (AUC 0.74 (95% CI 0.57–0.91), *p* = 0.013) and mixed rather than enteral nutrition (AUC 0.77 (95% CI 0.62–0.91), *p* = 0.005), were all strong predictors of a positive energy balance on day 14 (Figure 4B). The type of delivery, the presence of assisted reproduction, SGA, IUGR, and weight on admission did not influence the energy balance on day 14 (Supplemental Table S6). On day 7, macronutrients (*p* = 0.001) and enteral nutrition (*p* = 0.045) were also shown to predict a positive energy balance in the <34 weeks GA subcohorts.

#### 3.5.2. Predictors of a Positive Estimated Protein Balance

**Figure 5:**
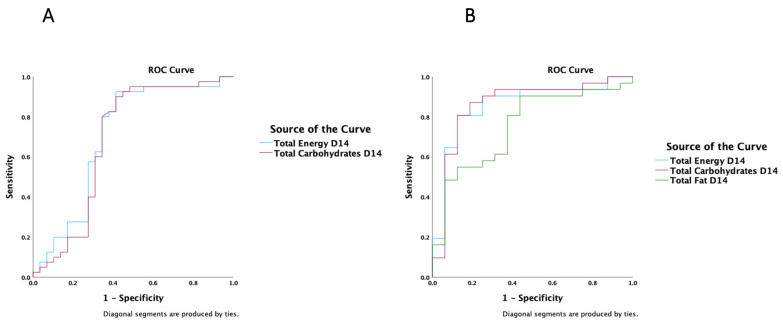
ROC analysis of a positive estimated protein balance on day 14 including (**A**) all gestational age (GA) subcohorts; (**B**) the smallest preterm infants with GA < 34 weeks.

In a ROC analysis regarding protein balance, total (enteral and parenteral) energy (AUC 0.70 (95% CI 0.57–0.84), *p* = 0.004) and carbohydrate (AUC 0.69 (95% CI 0.54–0.83), *p* = 0.009), but not fat, intakes on day 14 were strong predictors of a positive protein balance on day 14 (Figure 5A). The GA subcohorts, the presence of SGA, IUGR, sepsis, or surgery, no need for mechanical ventilation, the time until full enteral feeds, and the duration of parenteral nutrition did not influence the protein balance on day 14 (Supplemental Table S7). Cumulative carbohydrate provision (*p* = 0.023), enteral nutrition (*p* < 0.001), days until full enteral nutrition (*p* < 0.001), duration or parenteral nutrition (*p* < 0.001), and a small GA (*p* < 0.001) were all predictors of a positive protein balance on day 7.

When the ROC analysis was restricted to subcohorts with GA < 34 weeks, total (enteral and parenteral) energy (AUC 0.86 (95% CI 0.74–0.98), *p* < 0.001) carbohydrate (AUC 0.69 (95% CI 0.54–0.83), *p* = 0.009), and fat (AUC 0.75 (95% CI 0.61–0.90), *p* = 0.005) intakes on day 14 were strong predictors of a positive protein balance on day 14 (Figure 5B). The GA subcohorts, the presence of SGA, IUGR, sepsis, or surgery, no need for mechanical ventilation, the time until full enteral feeds, and the duration of parenteral nutrition did not influence the protein balance on day 14 (Supplemental Table S8). When ROC analysis was run on day 7, cumulative fat, rather than carbohydrate, provision (*p* = 0.049), along with enteral nutrition (*p* < 0.001), days until full enteral nutrition (*p* < 0.001), duration or parenteral nutrition (*p* < 0.001), and a small GA (*p* < 0.001) were shown to be strong predictors for a positive protein balance.

### 3.6. Nutrition and Growth

Days to regain birth weight differed significantly between GA subgroups (*p* < 0.001), with the extremely premature infants requiring the longest to regain birth weight (18.1 ± 4.5 days). On discharge, all anthropometric measurements differed among groups except head circumference z-scores (Table 7). GA subgroups showed negative Δ z-scores for weight. Extremely preterm and term infants had negative Δ z-scores in all anthropometry parameters. For the total population, only the difference in length z-scores was positive. The very-low birth weight preterm infants achieved the second better Δ z-score for length balance.

**Figure 6:**
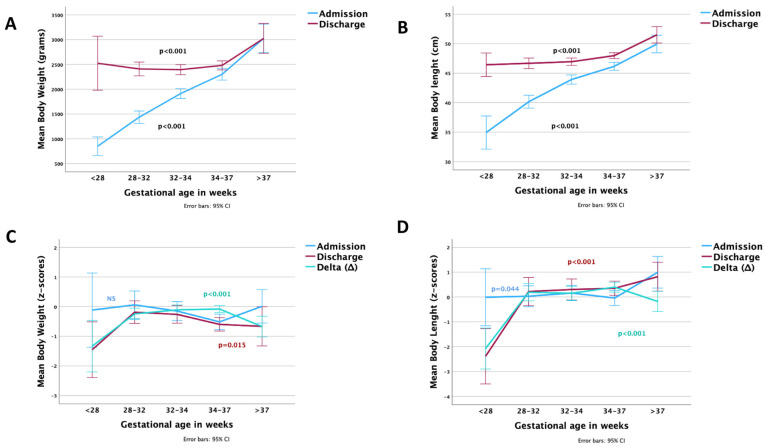
Comparable anthropometry at birth and discharge in different GAs: (**A**) body weight, (**B**) body length, (**C**), weight z-scores and delta z-scores, and (**D**) length z-scores and delta z-scores. The *p*-values express differences among all age groups at admission and discharge.

Despite having achieved comparable to the other groups’ body weight and length at discharge (Figure 6A,B), the extremely preterms (<28 weeks GA) had fallen to <the 5th percentile (<−1.65 z-score), which is the dreaded “EUGR” (Figure 6C,D). Weight gain velocity (g/kg BW/d) did not differ between sexes in any GA group or in the total cohort at discharge, but differed significantly among GA groups, with the highest values in the 28–34 weeks and the lowest in the >37 weeks GA (Table 7).

The negative Δ z-scores for body weight were significantly related to a negative protein balance on day 7 (*p* = 0.034) and day 14 (*p* = 0.008). Energy balance was more positive in patients with positive Δ z-scores on days 7 and 14 compared to those with negative Δ z-scores, but these differences did not reach statistical significance (Supplemental Figure S3).

### 3.7. Predictors of Growth

**Figure 7:**
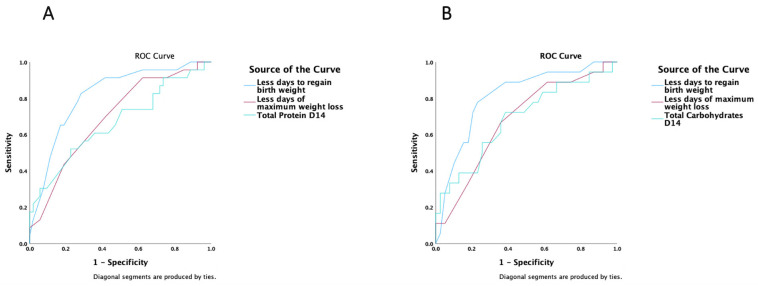
ROC analysis of a positive Δ z-score of weight at discharge in (**A**) all studied infants (**B**) in the <34 GA subcohorts.

In a ROC analysis, including all studied infants, only the total (enteral and parenteral) protein administered on day 14 (AUC 0.67 (95% CI 0.53–0.81), *p* = 0.022) was a strong predictor of a positive Δ z-score of weight at discharge (Figure 7A). Energy or specific substrates administered on either day 7 or 14, could not predict a positive Δ z-score of weight at discharge. A diagnosis of SGA (AUC 0.35 (95% CI 0.21–0.49), *p* = 0.041), the days to regain birth weight (AUC 0.18 (95% CI 0.08–0.26), *p* < 0.001), and the day of maximum weight loss (AUC 0.31 (95% CI 0.18–0.43), *p* = 0.007) were reverse predictors of a positive Δ z-score of weight at discharge (Figure 7A). The presence of IUGR, GA subcohorts, the time until full enteral feeds, and the duration of parenteral nutrition did not predict a positive Δ z-score of weight at discharge (Supplemental Table S9).

When the analysis was restricted to the <34 GA subcohorts, only total (enteral and parenteral) carbohydrates administered on day 14 (AUC 0.69 (95% CI 0.53–0.84), *p* = 0.025) could predict a positive Δ z-score of weight at discharge (Figure 7B). The days to regain birth weight (AUC 0.19 (95% CI 0.07–0.31), *p* < 0.001) and the day of maximum weight loss (AUC 0.32 (95% CI 0.17–0.47), *p* = 0.075) were reverse predictors of a positive Δ z-score of weight at discharge (Figure 7B). No energy or other substrates administered on either day 7 or 14, or any other nutritional, anthropometric, or clinical variable could predict a positive Δ z-score of weight at discharge in the <34 GA subcohorts (Supplemental Table S10).

### 3.8. Independent Associations with Catch up Growth 

To examine independent associations of nutritional and non-nutritional variables with a positive Δ z-score of weight at discharge, we ran a binary logistic regression analysis model (backward LR). When all GA subcohorts were included in the model, only a positive protein balance on day 14 (exp(B) 0.5 (95% CI 0.19–0.91), *p* = 0.028) and a short time to regain birth weight (exp(B) 1.5 (95% CI 1.2–1.8), *p* < 0.001) were independently associated with a positive Δ z-score of weight at discharge. In the <34 GA subcohorts, a positive protein balance on day 14 (exp(B) 0.9 (95% CI 0.8–1.0), *p* = 0.009) and a short time to regain birth weight (exp(B) 3.1 (95% CI 1.4–6.7), *p* = 0.004), were also independently associated with a positive Δ z-score of weight at discharge.

## 4. Discussion

In this retrospective research, we studied clinical and nutritional data for a cohort of infants of all gestational ages born and hospitalized in two separate NICUs. Based on minimum energy and protein requirements of <1800 g, preterm infants fed enterally by the ESPGHAN’s guidelines [25] and the ESPGHAN CoN recommendations for energy and protein intakes in the term and preterm infants, enterally and parenterally fed [34], we demonstrated that preterms beyond 28 weeks GA may achieve a positive estimated energy balance in the first week and a positive estimated protein balance in the second week in the NICU. Moreover, we showed that carbohydrate, fat, and protein intakes, a higher GA, and enteral nutrition were all strong predictors of a positive energy balance on days 7 and 14 for the whole cohort, including the <34 weeks GA subcohort. Similarly, energy, macronutrients, and early and sustained nutritional support could predict a positive protein balance on days 7 and 14. In our series, all GA subgroups exhibited negative Δ z-scores for weight on discharge, although those between 28 and 37 weeks had achieved positive Δ z-scores for length. Finally, we showed that a positive protein balance and the velocity of regaining birth weight were independently associated with catch-up growth in all gestational age groups, especially in the smallest infants.

The mean time interval from NICU admission to enteral feeding initiation day was 1.87 days for the whole population, while the extremely and the very premature neonates were started on days 3.5 and 2.6, respectively. While traditionally a more conservative approach with initiation of enteral feeds after the fourth day of life has been adopted in the past to minimize the risk of NEC [36], it is shown that more rapid and progressive enteral feeding starting from the first hours of life with minimal quantities is safe and does not increase the risk of NEC [37]. In our study, although EN was preferentially started within 24 or 48 h of life, and only if clinically not possible within 4 days, only one infant was diagnosed with NEC. Similarly, clinical implementation of evidence-based feeding protocols has been associated with improved growth without increased risk for NEC [38].

Extremely preterm infants needed significantly longer to achieve full enteral feeds, indicating the associated morbidities that potentially delay the advancement of enteral nutrition in this subgroup. Of note, in the very preterm subgroup, despite timely initiation of enteral feeding, time to full enteral feeds averaged 14 days. Among these infants, there were 9 infants with sepsis and the one infant with NEC. These clinical parameters have probably impaired the advancement of enteral feeds and delayed exclusive enteral nutrition. Two Cochrane Database systemic reviews could not determine whether the delayed or progressive introduction of enteral feeds affects growth and the risk of NEC in preterm or very low birth weight infants [39,40]. Accordingly, it is crucial that parenteral feeds are initiated on the first day of life to avoid a negative energy and protein balance. Despite the publication of guidelines and protocols, however, there is still great variability in clinical practice, considering the timing and composition of parenteral feeds [41]. In our series, parenteral nutrition was started on the first day of life for all infants < 32 weeks, usually combined with early minimal enteral nutrition. It has been shown that early minimal administration of enteral nutrition is associated with fewer parenteral nutrition-related metabolic side effects and higher survival rates [42].

Cumulative energy and macronutrients provided through the enteral and parenteral route were escalated longitudinally and differed among age groups during the study period and between days in all preterm groups. However, extremely premature infants presented negative energy balance on Days 7 and 14, despite achieving a positive protein balance. Martin et al., in a large multicentre prospective study of 1187 infants born < 28 weeks, showed that carbohydrate and total energy provision did not meet the published guidelines, while protein and fat approximated the recommendations [43]. In our series, carbohydrate, fat, and protein intakes, a higher GA, and enteral rather than parenteral nutrition, were all strong predictors of a positive estimated energy balance on day 14.

Infants in the very (28–31^+6^) and moderately (32–33^+6^) preterm groups maintained a positive energy balance but had opposite protein trends on day 7 and 14. In our series, no other nutritional, anthropometric, or clinical factor except for energy and substrate intakes could predict a positive protein balance on day 14. For the very preterm subgroup (28–31^+6^ weeks), the negative protein balance might reflect the transitional phase from parenteral to exclusively enteral feeding (mean day 14.1, Table 3) which is known to be a challenging period to achieve optimal intakes [25]. In a recent study, the length of the transition phase correlated negatively with cumulative energy and protein intakes at 28 days of age, and with weight and head circumference growth from birth to term equivalent age [15]. Parenteral nutrition can secure a positive protein intake, if prescribed according to guidelines. For enterally fed preterm neonates, consuming either unfortified human milk or regular infant formula deficient in protein affects the total protein intake. A mixed-cohort study showed that fortified human milk resulted in higher energy, fat, and carbohydrate intakes but a lower protein-to-energy ratio [21]. Similarly, enhanced parenteral nutrition in the very-low birth weight preterm infants resulted in increased energy intake and was feasible with no side effects [44]. In our study, the weight gain velocity was highest in the very preterm subgroup, obviously revealing an increased need for protein intake, which could not be achieved in this subcohort. However, the very low birth weight preterm infants achieved the second better Δ z-score for length balance, supporting the hypothesis that body length z-scores better reflect the skeletal growth and fat-free mass than the body weight z-scores [21], composed of organs and tissues growth [45].

Moderate preterm infants demonstrated the smallest deviations in protein balances. In contrast, late preterms consistently had a negative protein balance. In a recent study by Gerritsen at al., only 58% of moderate preterms and 19% of late preterms were prescribed the recommended protein intake [46]. There is growing evidence that nutritional management of late preterm infants poses more challenges. Frequently the same in size and weight as full terms, these infants can be falsely treated by caregivers as though they are physiologically mature [47]. However, they face issues related to their immaturity, such as weak sucking movements, lack of glycogen stores, and low breastmilk supply during their first days of life [48]. Although protein concentration in human milk correlates with the body weight gain of premature infants [49], breast milk content is highly variable between individuals of different postnatal age and gestational stage [50]. Highlighting the fact that nutritional guidelines for late and moderate preterm infants are absent, the ESPGHAN supports the use of breastmilk as the optimal nutrition for this category of infants, emphasizing the possible need for nutrient fortification in certain circumstances [51]. In our population, no infants of the late preterm group received fortified human milk.

Despite the positive length Δ z-scores in this study, all GA subcohorts showed negative Δ z-scores for weight at discharge, indicating some level of growth restriction, which was more evident in the extremely preterm subgroup. Whereas body length indicates fat-free mass and skeletal growth, body weight reflects tissues and organs’ weight [45]. In our study, the extremely preterm subcohort’s mean discharge weight z-score was −1.46, approaching percentiles below the 5th and meeting the definition of EUGR. Of note, these infants get discharged at later postmenstrual ages, often beyond the 40th week, so despite their weight at discharge being comparable with the other groups, their weight for age falls in lower curves. In accordance with our results, EUGR infants received 15% lower total energy and 35% lower protein intake on the seventh day of life compared to adequate growth infants [52]. Although the definition of EUGR indicating the negatively affected neonatal growth is considered a misnomer [53], discharge weight below the 10th percentile for age or drop in z-score > 1 SD are considered two acceptable definitions in the literature [54]. Obviously, new challenging standards are needed to be evaluated against currently used ones before they are implemented, and further studies should be conducted to evaluate the functional impact of such arbitrary cut-offs on long-term outcomes [4,55]. A recent large multicentre study reported a higher incidence of EUGR in non-SGA, extremely preterm infants compared with GAs > 28 weeks [56]. During the first days of life, neonates show some degree of weight loss that reflects a loss of extracellular fluid as they transition from the intrauterine to the extrauterine environment. Poor balance of this water homeostasis has been implicated in the later development of bronchopulmonary dysplasia and intraventricular haemorrhage [57]. In our population, maximum weight loss was generally noted between the 4th and 5th day of life, with extremely premature infants demonstrating a small delay.

Time to regain birth weight has been shown to be a predictive factor for postnatal growth velocity [58,59]. In our population, negative growth outcomes on discharge were related to a delay in the days to regain birth weight, a diagnosis of SGA, and a negative protein balance. Similar results arose from a recent observational study by Baillat et al., who demonstrated a negative effect of inadequate energy and protein intake in weight z-score and EUGR in moderately preterm infants [52]. Other randomized studies showed that feeding preterm infants enriched formulas had only modest effects on their growth rates [60]. In the study by Gerritsen et al., reaching the recommended protein intake was associated with a positive weight z-score difference at term age [46]. Importantly, in an early progressive enteral feeding with human milk of very low-birth-weight infants, carbohydrates, protein, and energy intakes correlated positively with weight gain and head circumference growth [59]. In a meta-analysis by Moyses et al., early parenteral nutrition was associated with improved short term growth outcomes such as the time to regain birth weight and weight at discharge [61]. In our cohort, the duration of parenteral nutrition could not independently predict a positive Δ z-score of weight at discharge, whereas the percentage of exclusive human milk feeding was low. Infants fed fortified human milk, based on its measured content, were discharged with significantly better weight gain, length, and head growth [21]. In our <34 weeks GA subcohorts, besides the days to regain birth weight and the day of maximum weight loss, total carbohydrates administered on day 14 could also predict a positive Δ z-score of weight at discharge. Based on GA, weight, day of life, different growing phases, and the ratio between enteral and parenteral intake, a computer-aided nutrition calculation program was shown to better achieve postnatal growth in the <32 GA preterms [60].

In our group of preterm infants of <34 weeks GA, a positive protein balance in the second week and an early regaining of birth weight independently improved catch-up growth at discharge. Supporting our results, randomized, controlled, double-blinded clinical trials have shown that a preterm formula with a higher protein: energy ratio could improve catch-up weight gain of preterm infants < 34 GA [62]. In addition, an enhanced protein diet demonstrated a more pronounced catch-up percentage of fat accretion in preterm infants of <32 GA with a lower baseline fat mass percentage [63]. The available data, however, are inadequate to draw conclusions on the effect of high protein feeds on safety and clinical outcomes [64]. Feeding preterms with formula, compared with donor breast milk, resulted in higher rates of weight gain but a higher risk of developing necrotising enterocolitis [65].

Certain limitations of this study should be acknowledged. Our observational study relied on retrospective collection and analysis of data and is prone to the pertinent biases. We aimed to mitigate this by pooling data from two independent centres and using a uniform data collection form. Our results indicate that early achievement of a positive energy –protein balance is crucial to prevent extrauterine growth restriction; however, it is a two-centre study with a relatively small sample size, and we consider this to be another limitation of this research. However, this is an original study comparing between and within separate GA subcohorts with a sample size well larger than the one of similar studies [21,66,67]. Although a third limitation could be the presented collected intake data on 3 days only, we have collected all days’ data of all 174 patients as is indicated in Table 3 and Table 4. However, analysis among five GA groups of everyday nutritional data was shown to be impractical, not producing any significant results compared with the three different weeks comparisons (3, 7, and 14 days), which revealed important findings, the same way similar studies had analysed their data between only these three days contributing to the literature [68,69]. We best considered anthropometrics in evaluating preterm infants’ growth by calculating and using weight, length, and head circumference Δ z-scores.

Our findings help bridge the recommended guidelines with their realistic implementation in daily practice and highlight specific difficulties. Similar studies serve as feedback and showcase the fields where guidelines’ goals are hardly achievable, leaving room for improvement to optimize clinical practice.

## 5. Conclusions

Results of this study showed that, while energy provision met the current European guidelines, protein provision was occasionally inadequate, leading to a negative protein balance. The latter seemed to have a detectable influence on weight at discharge. Early achievement of a positive energy and protein balance is crucial to ensure optimal postnatal growth and prevent extrauterine growth restriction, a relatively common occurrence in preterm infants. Late preterm infants seem to pose additional nutritional challenges and need individualized guidelines.

## Figures and Tables

**Table 1:** Average energy and protein content of preterm and term human milk and formulae.

	Preterm Formula/Human Milk	Infant Formula/Mature Human Milk
Energy (kcal/100 mL) [33]	73.5	67.5
Protein (g/100 mL) [33]	2.05	1.23

**Table 2:** ESPGHAN CoN recommendations for enteral nutrient intakes and ESPGHAN/ESPEN/ESPR/CSPEN Guidelines for preterm infants.

	Preterm Infants	Term Neonates
Energy (kcal/kg body weight/d)		
Enteral [25] *	115–140 (−160) **	90–120
Parenteral [35]	90–120	75–85
Protein (g/kg body weight/d)		
Enteral [25]	3.5–4.5	2.0–3.5
Parenteral [35]	2.5–3.5	2.0–3.0

* Recommended for <1800 g preterms. Previous recommendations for critically ill neonates were at a rate of 110–135 kcal/kg/d including all age groups [34]. ** Energy intakes >140 kcal/kg/d may be needed where growth is below the recommended range but should not be provided until protein and other nutrient sufficiency has been ensured and should not exceed 160 kcal/kg/d.

**Table 3:** Anthropometry and clinical data per gestational age group.

Patients’ Data	Gestational Age Groups						
	<28	28–31^+6^	32–33^+6^	34–36^+6^	>37	Total	*p*-Value
Neonates, *n* (%)	8 (4.6)	21 (12.1)	52 (29.9)	77 (44.3)	16 (9.2)	174 (100)	
Boys	4 (50)	10 (47.6)	36 (69.2)	45 (58.4)	12 (75)	107 (61.5)	0.287
Girls	4 (50)	11 (52.4)	16 (30.8)	32 (41.6)	4 (25)	67 (38.5)	
Anthropometry on admission, mean ± SD							
Birthweight (kg)	0.8 ± 0.22	1.43 ± 0.27	1.91 ± 0.36	2.3 ± 0.52	3.03 ± 0.54	2.08 ± 0.65	<0.001
Birthweight z-score *	−0.12 ± 1.49	0.05 ± 1.02	−0.15 ± 1.13	−0.51 ± 1.15	0.007 ± 1.06	−0.27 ± 1.15	0.143
Length (cm)	34.9 ± 3.3	40.1 ± 2.4	43.9 ± 2.7	46.1 ± 2.9	49.9 ± 2.7	44.5 ± 4.3	<0.001
Length z-score *	−0.01 ± 1.37	0.02 ± 0.92	0.15 ± 1.09	−0.04 ± 1.33	0.99 ± 1.19	0.12 ± 1.23	0.044
Head Circumference (cm)	23.6 ± 1.5	27.7 ± 1.4	30.4 ± 1.7	32.1 ± 1.7	34.1 ± 1.7	30.8 ± 2.8	<0.001
Head Circumference z-score *	−0.38 ± 0.89	−0.10 ± 0.82	−0.002 ± 1.16	0.06 ± 1.24	0.67 ± 1.29	0.05 ± 1.17	0.200
SGA, *n* (%)	2 (25)	3 (14.3)	6 (11.5)	19 (24.7)	2 (12.5)	32 (18.4)	0.344
IUGR, *n* (%)	2 (25)	3 (14.3)	3 (5.8)	18 (23.4)	2 (12.5)	28 (16.1)	0.099
Clinical data							
Length of stay (d), mean ± SD	90.3 ± 30.2	42.2 ± 12.3	22.6 ± 10.9	13.78 ± 6.65	7.31 ± 3.97	22.78 ± 20.65	<0.001
Resuscitation, *n* (%)	7 (87.5)	13 (61.9)	18 (34.6)	18 (23.4)	4 (25)	60 (34.5%)	<0.001
CRIB-II scores, mean ± SD	11 ± 1.77	4.11 ± 2.24	3.14 ± 2				
RDS, *n* (%)	7 (87.5)	11 (52.4)	23 (46)	27 (35.5)	11 (73.3)	79 (46.5)	0.004
Respiratory support, *n* (%total)	7 (87.5)	20 (95.2)	32 (61.5)	25 (32.5)	8 (50)	92 (52.9)	<0.001
Invasive MV, *n* (%supp)	7 (100)	14 (66.7)	13 (25)	5(6.5)	0	39 (42)	
NCPAP	0 (0)	5 (23.8)	17 (32.7)	19 (24.7)	6 (37.5)	47 (51)	
HFNC	0	1	1	1	2	5 (5)	
Diffuse O_2_	0	0	1	0	0	1 (1)	<0.001
None	0	1	20	52	8	81	
Days on invasive MV, mean ± SD	17.2 ± 11	2.5 ± 3.6	1.03 ± 1.3	0.59 ± 1.7	0	2.45 ± 5.6	<0.001
BPD, *n* (%)	4 (50)	2 (9.5)	0 (0)	0 (0)	0 (0)	6 (3.5)	<0.001
Sepsis, *n* (%)	5 (71.4)	5 (23.8)	3 (5.8)	4 (5.2)	0 (0)	17 (9.8)	<0.001
PDA, *n* (%)	3 (42.9)	1 (4.8)	2 (3.9)	1 (1.3)	0 (0)	7 (4.1)	<0.001
NEC, *n* (%)	1 (14.3)	0 (0)	0 (0)	0 (0)	0 (0)	1 (0.6)	<0.001

SD = Standard Deviation; SGA= Small for gestational age; IUGR = Intrauterine Growth Restriction; CRIB = Clinical Risk Index for Babies; MV = Mechanical Ventilation; NCPAP = Nasal Continuous Positive Airway Pressure; HFNC = High-Flow Nasal Cannula; RDS = Respiratory Distress Syndrome; BPD = Bronchopulmonary Dysplasia; PDA = Patent Ductus Arteriosus, NEC = Necrotizing Enterocolitis. * All z-scores are extracted using INTERGROWTH-21st standard curves for newborn size at birth.

**Table 4:** Timings of Enteral Nutrition (EN) and Parenteral Nutrition (PN) across GA subgroups.

Patient Data	Gestational Age Groups						
	<28	28–31^+6^	32–33^+6^	34–36^+6^	>37	Total	*p* Value
Timing	Mean ± SD						
Day of EN initiation	3.5 ± 0.9	2.62 ± 2.08	1.94 ± 1.3	1.55 ± 1	1.33 ± 0.61	1.87 ± 1.3	<0.001
Days until full EN	54.7 ± 28.6	14.1 ± 6.2	5.51 ± 5.1	2.01 ± 2.8	0.75 ± 1.1	6.91 ± 13.4	<0.001
Day of PN initiation	1.0 ± 0.0	1.0 ± 0.0	1.6 ± 4.26	1.23 ± 0.74	1.0 ± 0	1.32 ± 2.6	0.857
Duration of PN	56.2 ± 25.1	13.9 ± 5.6	5.8 ± 4.5	3.54 ± 2.7	1.83 ± 0.98	9.75 ± 15.1	<0.001

SD = Standard Deviation; EN = Enteral Nutrition; PN = Parenteral Nutrition.

**Table 5:** Cumulative energy and protein from enteral and parenteral nutrition on Days 3, 7, and 14.

Patient Data	Gestational Age Groups						
	<28	28–31^+6^	32–33^+6^	34–36^+6^	>37	Total	*p* Value *
Total Energy (kcal/kg BW/d)	Mean ± SD						
Day 3	62.8 ± 13.4	68.2 ± 14.36	75.9 ± 18.8	85.4 ± 20.8	81.2 ± 16.3	79.2 ± 19.9	<0.001
Day 7	78.2 ± 29.7	94.9 ± 14.4	110.2 ± 23.2	116.4 ± 19.3	169.2 ± 92.9	111.9 ± 31.4	<0.001
Day 14	72.2 ± 28.3	102.9 ± 24.1	120.7 ± 27.9	137.3 ± 25.9	145.2 ± 36.7	120.2 ± 31.8	<0.001
*p* value **	0.006	<0.001	<0.001	<0.001	0.223	<0.001	
Total Protein (g/kg BW/d)	Mean ± SD						
Day 3	3.3 ± 0.5	3.5 ± 0.4	2.7 ± 0.9	2.0 ± 0.8	1.5 ± 0.4	2.4 ± 0.9	<0.001
Day 7	3.0 ± 1.2	3.6 ± 0.6	3.2 ± 0.9	2.7 ± 0.9	3.0 ± 1.7	3.1 ± 1.0	0.003
Day 14	3.1 ± 1.3	3.6 ± 0.5	3.6 ± 0.7	3.0 ± 0.8	2.6 ± 0.7	3.3 ± 0.8	0.012
*p* value **	0.244	0.437	<0.001	<0.001	0.223	<0.001	

BW = body weight. Group differences (*p* values): * ANOVA test; ** Related-Samples Friedman’s Two-Way Analysis of Variance by Ranks.

**Table 6:** Estimated energy and protein balances on Day 7 and 14 of different GA subgroups.

Patient Data	Gestational Age Groups						
	<28	28–31^+6^	32–33^+6^	34–36^+6^	>37	Total	*p* Value
Administered— Recommended difference	Mean ± SD						
Energy balance *, D7 (kcal/kg/d), mean ± SD	−1.64 ± 8.8	3.04 ± 12.2	6.05 ± 18.5	8.04 ± 18.7	79.1 ± 92.9	9.44 ± 29.2	<0.001
Energy balance, D14 (kcal/kg/d), mean ± SD	−26.7 ± 9.9	5.3 ± 20.4	14.5 ± 24.5	27.3 ± 25.8	55.2 ± 36.6	17.9 ± 27	<0.001
Protein balance **, D7 (g/kg/d), mean ± SD	0.92 ± 0.47	0.96 ± 0.73	−0.21 ± 0.86	−0.70 ± 0.92	1.07 ± 1.69	−0.10 ± 1.11	<0.001
Protein balance, D14 (g/kg/d), mean ± SD	0.66 ± 0.57	−0.09 ± 0.83	0.06 ± 0.76	−0.45 ± 0.81	0.63 ± 0.66	−0.13 ± 0.82	0.011

Estimated energy and protein balance calculations are based on ESPGHAN guidelines regarding the minimum energy * and protein ** requirements of enterally and parenterally fed preterm and term infants; SD = Standard Deviation.

**Table 7:** Discharge data: Anthropometry, Delta (Δ) z-score for anthropometry, weight gain velocity, days to regain birthweight, and days of maximum weight loss.

Patient Data	Gestational Age Groups						
	<28	28–31^+6^	32–33^+6^	34–36^+6^	>37	Total	*p* Value
Anthropometry on discharge *	Mean ± SD						
Bodyweight (kg)	2.50 ± 0.65	2.40 ± 0.30	2.39 ± 0.36	2.48 ± 0.40	3.02 ± 0.56	2.50 ± 0.44	<0.001
Bodyweight z-score	−1.46 ± 1.12	−0.19 ± 0.83	−0.27 ± 1.06	−0.60 ± 0.99	−0.67 ± 1.24	−0.49 ± 1.05	0.015
Length (cm)	46.4 ± 2.23	46.7 ± 1.96	46.9 ± 2.23	47.9 ± 2.17	51.5 ± 2.61	49.8 ± 2.55	<0.001
Length z-score	−2.39 ± 1.24	0.22 ± 1.25	0.30 ± 1.51	0.35 ± 1.26	0.822 ± 1.09	0.25 ± 1.43	<0.001
Head Circumference (cm)	34.4 ± 1.65	32.9 ± 1.58	32.7 ± 1.10	33.0 ± 1.22	34.4 ± 1.63	33.1 ± 1.36	<0.001
Head Circumference z-score	−0.78 ± 1.33	0.39 ± 1.30	0.15 ± 0.94	−0.09 ± 1.13	−0.01 ± 1.54	0.02 ± 1.16	0.145
Delta (Δ) z-Scores **	Mean ± SD						*p* value
Weight	−1.33 ± 1.03	−0.24 ± 0.42	−0.11 ± 0.59	−0.08 ± 0.51	−0.67 ± 0.65	−0.22 ± 0.64	<0.001
Length	−2.07 ± 0.98	0.18 ± 0.76	0.14 ± 1.0	0.39 ± 0.88	−0.18 ± 0.76	0.12 ± 1.03	<0.001
Head Circumference	−0.29 ± 1.07	0.49 ± 1.13	0.13 ± 0.71	−0.14 ± 0.85	−0.68 ± 0.76	−0.04 ± 0.9	<0.001
Weight gain velocity (g/kg BW/d)	Mean ± SD						*p* value
Boys	18.8 ± 1.29	23.5 ± 4.46	19.1 ± 13.8	6.85 ± 15.3	−10.8 ± 30.0	10.9 ± 18.8	<0.001
Girls	15.8 ± 6.31	20.9 ± 6.11	19.3 ± 11.7	11.5 ± 14.5	2.50 ± 12.4	14.6 ± 13.1	<0.035
Days to regain birth weight	18.1 ± 4.5	12.6 ± 4.2	10.4 ± 3.7	8.7 ± 3.7	6.6 ± 3.6	10.3 ± 4.5	<0.001
Day of maximum weight loss	7.5 ± 2.8	5.25 ± 1.5	4.1 ± 2.37	4.48 ± 2.17	3.93 ± 1.32	4.55 ± 2.2	<0.001

* At discharge, z-scores were extracted using INTERGROWTH21st standard curves for postnatal growth of premature infants. ** Delta (Δ) z-scores are the differences of the discharge–birth calculated z-scores for anthropometry; SD = Standard Deviation; BW = Body Weight.

## Data Availability

The datasets generated and analysed during the current study are not publicly available due the database being very extensive and including data from other studies complementary to this, but they are available from the corresponding authors upon reasonable request.

## Supplementary Materials

The following supporting information can be downloaded at: https://www.mdpi.com/article/10.3390/nu15163556/s1: Supplemental Figure S1. Subcohorts of neonates included in the study: A. Gestational Age distribution. B. Birth Weight distribution. Supplemental Figure S2. Correlation of gestational age with the length of stay. Supplemental Table S1. Composition of term and preterm milk formulas used in the two NICUs during the study period. Supplemental Table S2. Energy, protein, and carbohydrate content of powder human milk fortifiers used in the two NICUs during the study (2.2 g/sachet to be added to 50 mL HM). Supplemental Table S3. Concentrations of amino acid solution and lipid emulsion used in parenterally fed neonates in the NICU during the study period. Supplemental Figure S3. Schematic algorithm for initiating and advancing enteral feeding in preterm infants in the NICU. Supplemental Table S4. Cumulative energy and protein from enteral and parenteral nutrition on each consecutive Day from Day 3 to Day 14. Supplemental Table S5. Prediction ability of a positive energy balance on day 14 by anthropometric, nutritional, and clinical variables in all GA subcohorts. Supplemental Table S6. Prediction ability of a positive energy balance on day 14 by anthropometric, nutritional, and clinical variables in the smallest preterm subcohorts (GA < 34 weeks). Supplemental Table S7. Prediction ability of a positive protein balance on day 14 by anthropometric, nutritional, and clinical variables in all GA subcohorts. Supplemental Table S8. Prediction ability of a positive protein balance on day 14 by anthropometric, nutritional, and clinical variables in the smallest preterm subcohorts (GA < 34 weeks). Supplemental Figure S4. Relation of Δ weight z-score with energy and protein balance on Days 7 and 14. Supplemental Table S9. Prediction ability of a positive Δ z-score of weight at discharge by nutritional variables and anthropometry including all gestational age infants. Supplemental Table S10. Prediction ability of a positive Δ z-score of weight at discharge by nutritional variables and anthropometry in <34 gestational age preterm infants.
