# Supplementary material for: The Impact of Estimated Energy and Protein Balances on Extrauterine Growth in Preterm Infants
Source: Nutrients. 2023 Aug 11;15(16):3556. doi: 10.3390/nu15163556 (PMC10458304; doi:10.3390/nu15163556)
Supplement: Supplementary file 1 [file nutrients-15-03556-s001.zip › nutrients-2553878-supplementary.pdf]

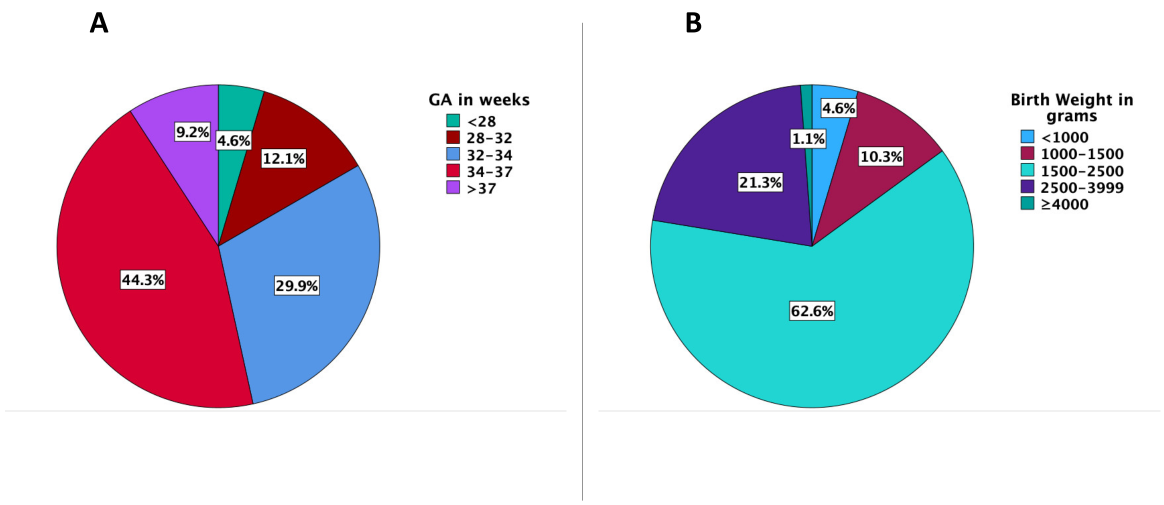

Supplemental Figure S1. Sub cohorts of neonates included in the study: A. Gestational Age distribution. B. Birth Weight distribution.

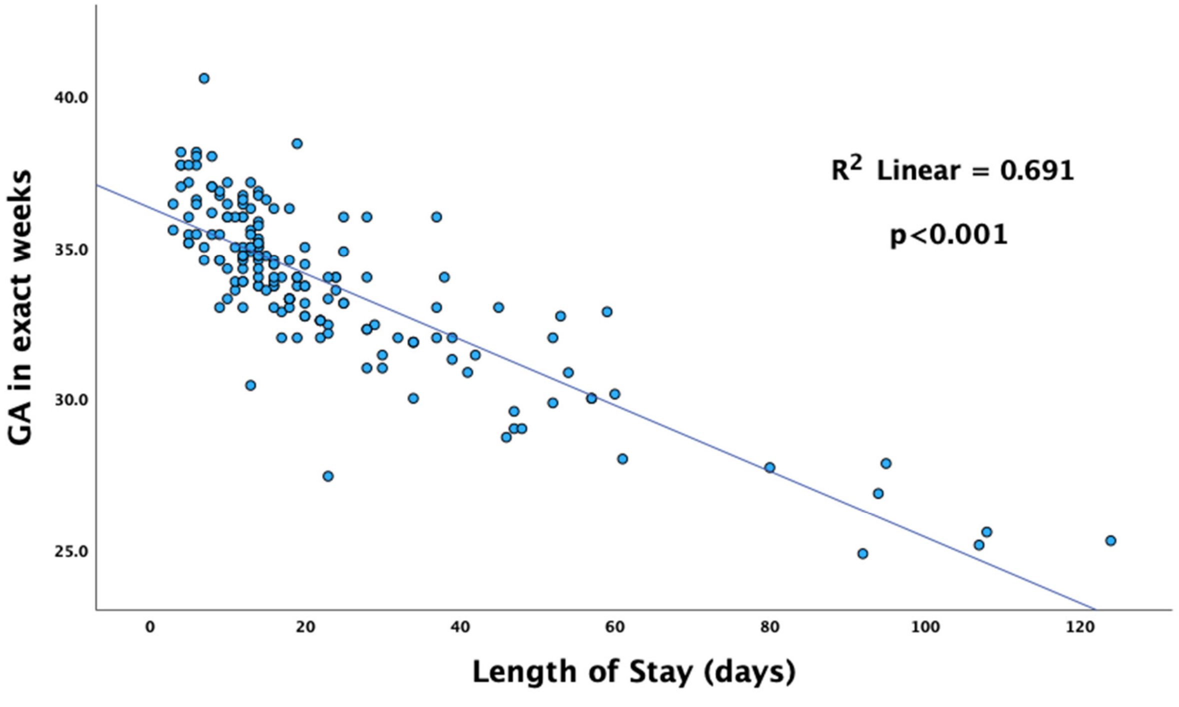

Supplemental Figure S2. Correlation of gestational age with the length of stay.

Supplemental Table S1. Composition of term and preterm milk formulas used in the two NICUs during the study period.

	Term Formula	Preterm Formula
Energy (kcal/100ml)	66	80
Carbohydrates (g/100ml)	7.4	8.3
Protein (g/100ml)	1.3	2.7
Lipids (g/100ml)	3.4	3.9
Sodium (mg)	25	70
Potassium (mg)	88	81
Chloride (mg)	54	86
Calcium (mg)	50	101
Phosphate (mg)	37	63
Magnesium (mg)	5.3	7.4
Iron (mg)	0.53	1.6
Zinc (mg)	0.51	1.1
Copper (mg)	0.052	0.08
Manganese (mg)	0.003	0.006
Iodine (μg)	13	27
Selenium (μg)	3	4.5
Vitamin A (μg)	58	366
Vitamin D3 (μg)	1.7	3.1
Vitamin E (mg)	1.1	4.6
Vitamin K (μg)	4.1	6.7
Vitamin C (mg)	9.2	18
Thiamine (mg)	0.07	0.14
Riboflavin (mg)	0.14	0.20
Niacin (mg)	0.43	3.2
Vitamin B12 (μg)	0.13	0.2
Folic Acid (μg)	13	35

Supplemental Table S2. Energy, protein and carbohydrate content of powder human milk fortifiers used in the two NICUs during the study (2.2g/sachet to be added to 50ml HM)

	/100gr	/Sachet (2.2gr)
Energy (kcal)	347	6.94
Carbohydrates (g)	62.2	1.36
Protein(g)	25.2	0.55
Fat (g)	0	0

Supplemental Table S3. Concentrations of amino acid solution and lipid emulsion used in parenterally fed neonates in the NICU during the study period.

Amino Acid solution	g/1000ml	Lipid Emulsion	g/1000ml
L-Alanine	6.3	Soybean oil	60
L-Arginine	4.1	Medium chain TGs	60
L-Aspartic	4.1	Olive oil	50
Cysteine hydrochloride	1	Fish oil (Ω3 PUFAs, EPA, DHA)	30
Glutamic Acid	7.1		
Amino acetic acid	2.1		
L-histidine	2.1		
L-Isoleucine	3.1		
L-Leucine	7		
L-Lysine	5.6		
Methionine	1.3		
Phenylalanine	2.7		
Proline	5.6		
L-serine	3.8		
Taurine	3		
L-Threonine	3.6		
L-Tryptophane	1.4		
L-Tyrosine	0.5		
L-valine	3.6		

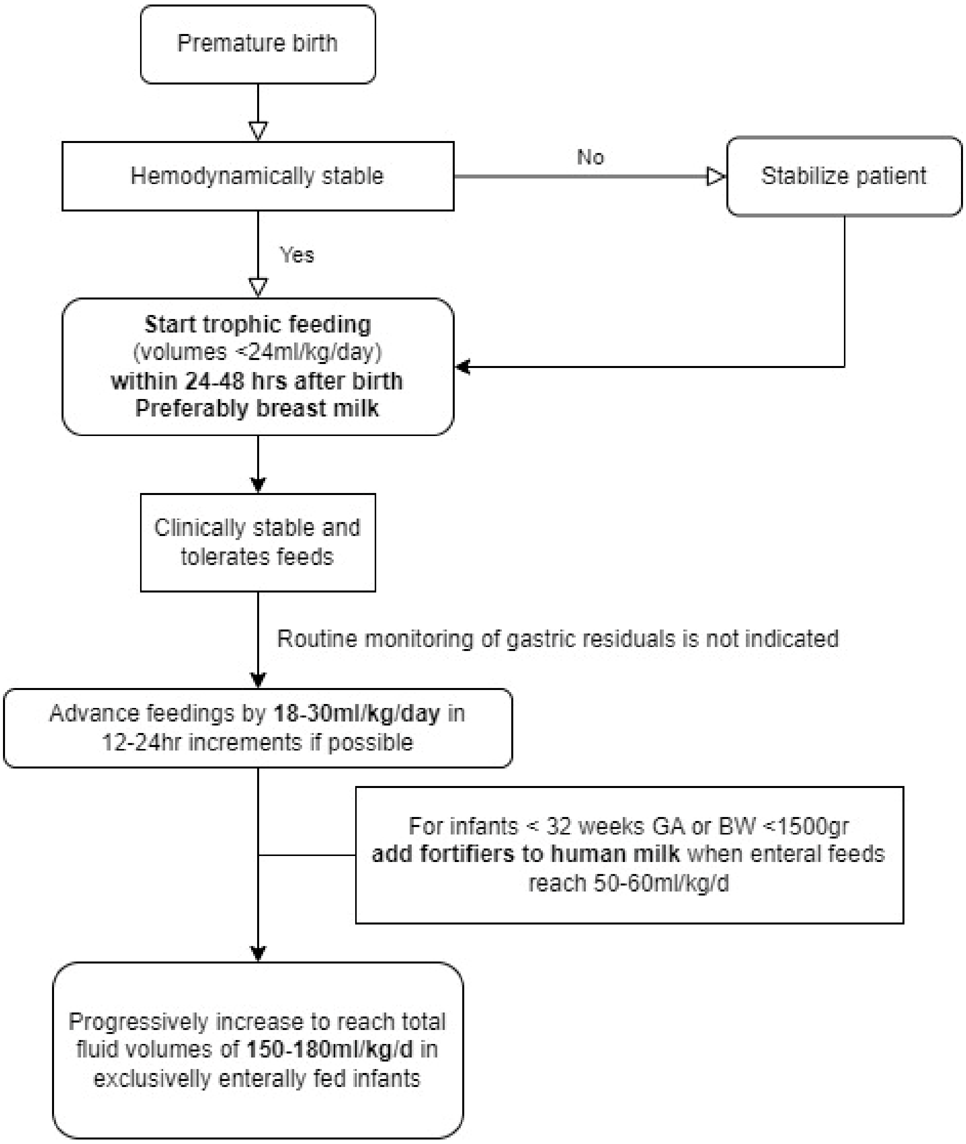

Supplemental Figure S3. Schematic algorithm for initiating and advancing enteral feeding in preterm infants in the NICU.

Supplemental Table S4. Cumulative energy and protein from enteral and parenteral nutrition on each consecutive Day from Day 3 to Day 14.

Patient data	Gestational Age Groups						
	<28	28-31^{+6}	32-33^{+6}	34-36^{+6}	>37	Total	*p* value*
Total Energy (kcal/kg BW/d)	Mean±SD						
Day 3	62.8±13.4	68.2±14.4	75.9±18.8	85.4±20.8	81.2±16.3	79.2±19.9	<0.001
Day 4	67.1±10.1	76.4±17.2	84.3±15.5	93.2±17.1	95.7±18.1	87.6±18.1	<0.001
Day 5	71.4±14.6	81.0±10.6	93.2±16.9	101.4±18.8	120.0±19.3	94.2±23.4	<0.001
Day 6	75.0±11.2	87.6±13.5	102.1±14.1	109.9±20.0	143.8±19.4	103.1±26.7	<0.001
Day 7	78.2±29.7	94.9±14.4	110.2±23.2	116.4±19.3	169.2±92.9	111.9±31.4	<0.001
Day 8	70.8±11.0	95.1±13.5	108.9±18.3	119.2±17.2	170.4±16.8	104.1±19.1	<0.001
Day 9	66.4±15.0	95.6±14.2	109.3±15.8	121.3±16.7	171.8±19.9	105.6±29.2	<0.001
Day 10	62.8±13.4	97.2±12.2	111.8±16.6	125.9±19.9	164.7±19.3	108.4±23.4	<0.001
Day 11	63.9±09.8	98.9±15.0	113.2±20.1	128.4±21.1	162.6±32.0	115.9±33.5	<0.001
Day 12	65.7±11.1	100.1±11.8	115.9±18.0	131.4±22.2	156.2±26.2	117.0±29.3	<0.001
Day 13	69.3±12.8	101.7±16.2	118.4±19.5	134.9±23.6	151.8±28.9	119.4±23.6	<0.001
Day 14	72.2±28.3	102.9±24.1	120.7±27.9	137.3±25.9	145.2±36.7	120.2±31.8	<0.001
p value**	0.008	<0.01	<0.001	<0.001	0.301	<0.001	
Total Protein (g/kg BW/d)	Mean±SD						
Day 3	3.3±0.5	3.5±0.4	2.7±0.9	2.0±0.8	1.5±0.4	2.4±0.9	<0.001
Day 4	3.0±1.2	3.6±0.6	2.9±0.6	2.7±0.9	3.0±1.7	3.1±1.0	<0.001
Day 5	3.0±1.2	3.6±0.6	3.0±0.9	2.7±0.9	3.0±1.7	3.1±1.0	<0.001
Day 6	3.0±1.2	3.6±0.6	3.1±0.8	2.7±0.9	3.0±1.7	3.1±1.0	0.004
Day 7	3.0±1.2	3.6±0.6	3.2±0.9	2.7±0.9	3.0±1.7	3.1±1.0	0.003
Day 8	3.0±1.3	3.6±0.5	3.2±0.8	2.7±0.8	2.9±0.7	3.1±0.8	<0.001
Day 9	3.0±1.1	3.6±0.5	3.3±0.8	2.8±0.8	2.9±0.7	3.1±0.9	<0.001
Day 10	3.0±1.6	3.6±0.6	3.4±0.7	2.9±0.9	2.8±0.8	3.2±0.9	0.002
Day 11	3.1±1.1	3.6±0.7	3.4±0.7	2.9±0.8	2.7±0.7	3.2±0.8	<0.001
Day 12	3.1±1.1	3.7±0.5	3.5±0.6	3.0±0.9	2.7±0.9	3.3±1.0	<0.001
Day 13	3.1±1.2	3.6±0.7	3.6±0.8	3.0±0.9	2.6±0.8	3.3±0.9	<0.001
Day 14	3.1±1.3	3.6±0.5	3.6±0.7	3.0±0.8	2.6±0.7	3.3±0.8	0.012
p value**	0.398	0.687	<0.001	<0.001	0.431	<0.001	

Supplemental Table S5. Prediction ability of a positive energy balance on day 14 by anthropometric, nutritional, and clinical variables in all GA sub cohorts.

Area Under the Curve					
Test Result Variable(s)	Area	Std. Error	*p* value	Asymptotic 95% Confidence Interval	
				Lower Bound	Upper Bound
Total Fat Day 14	0.994	0.007	0.000	0.981	1.000
Total Carbohydrates Day 14	0.955	0.035	0.000	0.888	1.000
Total Protein Day 14	0.801	0.056	0.000	0.692	0.910
SGA	0.419	0.072	0.307	0.278	0.560
IUGR	0.431	0.073	0.387	0.288	0.575
Nutritional Support on Day 14 *	0.406	0.086	0.238	0.238	0.574
GA in exact weeks	0.723	0.073	0.005	0.579	0.867
Weight on Admission (grams)	0.536	0.092	0.655	0.355	0.716
Type of Delivery	0.569	0.083	0.387	0.406	0.732
Assisted Reproduction	0.481	0.079	0.813	0.326	0.636
Nutritional Support on Day 7 *	0.269	0.072	0.004	0.127	0.411

GA = Gestational age; SGA= Small for gestational age; IUGR = Intrauterine Growth Restriction.
* Enteral vs. Parenteral vs. Enteral and Supplemental Parenteral.

Supplemental Table S6. Prediction ability of a positive energy balance on day 14 by anthropometric, nutritional, and clinical variables in the smallest preterm sub cohorts (GA < 34 weeks).

Test Result Variable(s)	Area	Std. Error	*p* value	Asymptotic 95% Confidence Interval	
				Lower Bound	Upper Bound
Total Fat Day 14	0.988	0.012	0.000	0.964	1.000
Total Carbohydrates Day 14	0.922	0.059	0.000	0.806	1.000
Total Protein Day 14	0.870	0.049	0.000	0.775	0.966
SGA	0.472	0.093	0.768	0.290	0.653
IUGR	0.484	0.094	0.862	0.300	0.667
Nutritional Support on Day 14 *	0.375	0.102	0.189	0.176	0.574
GA in exact weeks	0.737	0.086	0.013	0.569	0.906
Weight on Admission (grams)	0.616	0.099	0.221	0.422	0.810
Type of Delivery	0.567	0.099	0.482	0.374	0.760
Assisted Reproduction	0.481	0.094	0.839	0.296	0.666
Nutritional Support on Day 7 *	0.234	0.075	0.005	0.087	0.382

GA = Gestational age; SGA= Small for gestational age; IUGR = Intrauterine Growth Restriction.
* Enteral vs. Parenteral vs. Enteral and Supplemental Parenteral.

Supplemental Table S7. Prediction ability of a positive protein balance on day 14 by anthropometric, nutritional, and clinical variables in all GA sub cohorts.

Area Under the Curve					
Test Result Variable(s)	Area	Std. Error	*p* value	Asymptotic 95% Confidence Interval	
				Lower Bound	Upper Bound
Total energy Day 14	0.704	0.070	0.004	0.567	0.842
Total Fat Day 14	0.626	0.073	0.076	0.483	0.768
Total Carbohydrates Day 14	0.685	0.074	0.009	0.540	0.830
GA in exact weeks	0.374	0.070	0.075	0.237	0.511
SGA	0.409	0.068	0.202	0.276	0.543
IUGR	0.439	0.069	0.391	0.303	0.575
Days until full enteral feeds	0.540	0.073	0.576	0.397	0.682
Duration of PN	0.498	0.073	0.976	0.354	0.641
Surgery	0.517	0.071	0.808	0.378	0.657
Sepsis	0.502	0.071	0.981	0.363	0.641
Non-Invasive Respiratory Support	0.538	0.071	0.589	0.400	0.677

GA = Gestational age; SGA= Small for gestational age; IUGR = Intrauterine Growth Restriction; PN= parenteral nutrition.

Supplemental Table S8. Prediction ability of a positive protein balance on day 14 by anthropometric, nutritional, and clinical variables in the smallest preterm sub cohorts (GA < 34 weeks).

Area Under the Curve					
Test Result Variable(s)	Area	Std. Error	*p* value	Asymptotic 95% Confidence Interval	
				Lower Bound	Upper Bound
Total energy Day 14	0.857	0.062	0.000	0.736	0.978
Total Fat Day 14	0.752	0.075	0.005	0.605	0.899
Total Carbohydrates Day 14	0.861	0.065	0.000	0.734	0.987
GA in exact weeks	0.489	0.091	0.902	0.311	0.666
SGA	0.482	0.089	0.840	0.307	0.656
IUGR	0.498	0.090	0.982	0.322	0.674
Days until full enteral feeds	0.466	0.097	0.703	0.275	0.657
Duration of PN	0.445	0.094	0.537	0.260	0.629
Surgery	0.531	0.091	0.728	0.352	0.710
Sepsis	0.514	0.090	0.875	0.337	0.691
Non-Invasive Respiratory Support	0.460	0.094	0.653	0.276	0.643

GA = Gestational age; SGA= Small for gestational age; IUGR = Intrauterine Growth Restriction; PN= parenteral nutrition.

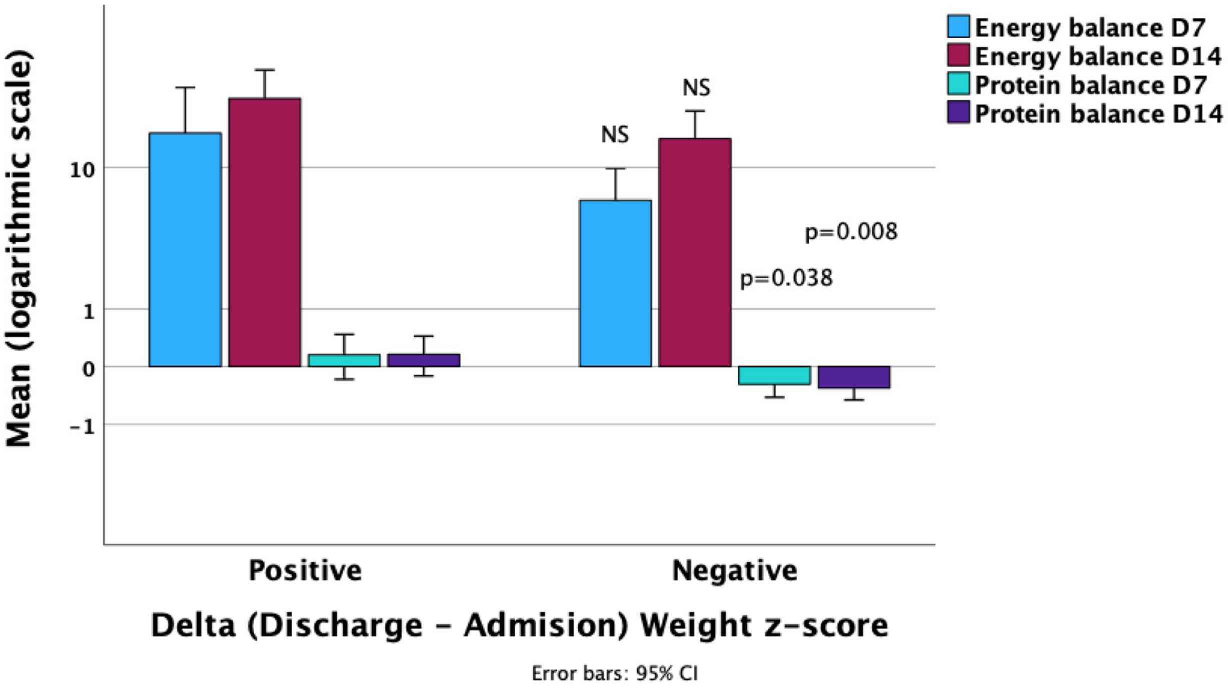

Supplemental Figure S4. Relation of Δ weight z-score with energy and protein balance on Days 7 and 14.

Supplemental Table S9. Prediction ability of a positive Δ z-score of weight at discharge by nutritional variables and anthropometry including all gestational age infants.

Area Under the Curve					
Test Result Variable(s)	Area	Std. Error	p value	Asymptotic 95% Confidence Interval	
				Lower Bound	Upper Bound
Total energy Day 7	0.517	0.073	0.817	0.373	0.661
Total energy Day 14	0.578	0.074	0.283	0.434	0.722
Total Protein Day 7	0.532	0.070	0.663	0.393	0.670
Total Protein Day 14	0.666	0.071	0.022	0.526	0.806
Total Fat Day 7	0.498	0.069	0.977	0.362	0.634
Total Fat Day 14	0.559	0.074	0.412	0.415	0.704
Total Carbohydrates Day 7	0.482	0.075	0.799	0.334	0.629
Total Carbohydrates Day 14	0.624	0.069	0.087	0.490	0.759
GA in exact weeks	0.493	0.070	0.928	0.357	0.630
SGA	0.352	0.074	0.041	0.207	0.496
IUGR	0.457	0.074	0.557	0.313	0.602
Days until full enteral feeds	0.469	0.070	0.672	0.332	0.606
Duration of PN	0.433	0.071	0.354	0.293	0.572
Days to regain birth weight	0.183	0.052	0.000	0.081	0.285
Day of maximum weight loss	0.305	0.064	0.007	0.179	0.431

GA = Gestational age; SGA= Small for gestational age; IUGR = Intrauterine Growth Restriction; PN= parenteral nutrition.
*Smaller test result indicates more positive test (inverse relations: 1-AUC).

Supplemental Table S10. Prediction ability of a positive Δ z-score of weight at discharge by nutritional variables and anthropometry in < 34 gestational age preterm infants.

Area Under the Curve					
Test Result Variable(s)	Area	Std. Error	p value	Asymptotic 95% Confidence Interval	
				Lower Bound	Upper Bound
Total energy Day 7	0.498	0.086	0.979	0.330	0.666
Total energy Day 14	0.604	0.087	0.210	0.434	0.774
Total Protein Day 7	0.465	0.085	0.674	0.298	0.633
Total Protein Day 14	0.563	0.093	0.445	0.382	0.745
Total Fat Day 7	0.524	0.080	0.770	0.367	0.682
Total Fat Day 14	0.601	0.086	0.223	0.432	0.770
Total Carbohydrates Day 7	0.454	0.087	0.577	0.283	0.624
Total Carbohydrates Day 14	0.687	0.079	0.025	0.532	0.841
GA in exact weeks	0.528	0.078	0.731	0.375	0.682
SGA	0.412	0.085	0.291	0.246	0.579
IUGR	0.496	0.083	0.959	0.333	0.659
Days until full enteral feeds	0.405	0.080	0.254	0.248	0.562
Duration of PN	0.390	0.081	0.186	0.231	0.550
Days to regain birth weight	0.191	0.062	0.000	0.070	0.312
Day of maximum weight loss	0.320	0.075	0.030	0.172	0.467

GA = Gestational age; SGA= Small for gestational age; IUGR = Intrauterine Growth Restriction; PN= parenteral nutrition.
